# Real-Time Sensor Data Profile-Based Deep Learning Method Applied to Open Raceway Pond Microalgal Productivity Prediction

**DOI:** 10.1021/acs.est.2c07578

**Published:** 2023-05-26

**Authors:** Thomas Igou, Shifa Zhong, Elliot Reid, Yongsheng Chen

**Affiliations:** † School of Civil & Environmental Engineering, Georgia Institute of Technology, Atlanta, Georgia 30332, United States; ‡ Department of Environmental Science, School of Ecological and Environmental Sciences, East China Normal University, Shanghai 200241, PR China

**Keywords:** open raceway pond, microalgae productivity, machine learning, deep learning, algal biofuels

## Abstract

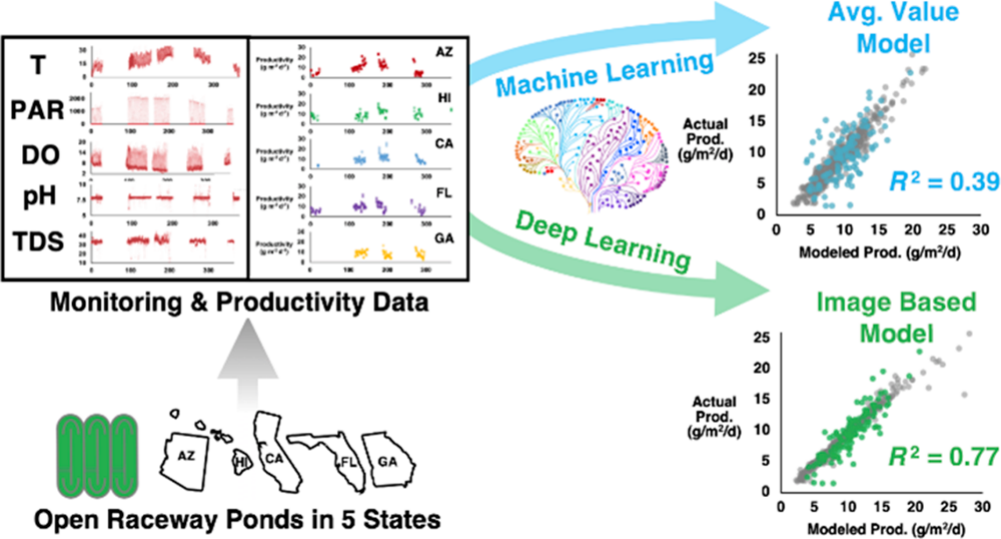

Microalgal biotechnology holds the potential for renewable biofuels, bioproducts, and carbon capture applications due to unparalleled photosynthetic efficiency and diversity. Outdoor open raceway pond (ORP) cultivation enables utilization of sunlight and atmospheric carbon dioxide to drive microalgal biomass synthesis for production of bioproducts including biofuels; however, environmental conditions are highly dynamic and fluctuate both diurnally and seasonally, making ORP productivity prediction challenging without time-intensive physical measurements and location-specific calibrations. Here, for the first time, we present an image-based deep learning method for the prediction of ORP productivity. Our method is based on parameter profile plot images of sensor parameters, including pH, dissolved oxygen, temperature, photosynthetically active radiation, and total dissolved solids. These parameters can be remotely monitored without physical interaction with ORPs. We apply the model to data we generated during the Unified Field Studies of the Algae Testbed Public-Private-Partnership (ATP^3^ UFS), the largest publicly available ORP data set to date, which includes millions of sensor records and 598 productivities from 32 ORPs operated in 5 states in the United States. We demonstrate that this approach significantly outperforms an average value based traditional machine learning method (*R*^2^ = 0.77 ≫ *R*^2^ = 0.39) without considering bioprocess parameters (e.g., biomass density, hydraulic retention time, and nutrient concentrations). We then evaluate the sensitivity of image and monitoring data resolutions and input parameter variations. Our results demonstrate ORP productivity can be effectively predicted from remote monitoring data, providing an inexpensive tool for microalgal production and operational forecasting.

## Abstract 2

Development of new image-based deep learning methodology to forecast outdoor open raceway pond microalgal productivity with inexpensive sensors.

## 1 Introduction

Microalgae are a diverse collection (e.g., >30,000 species) of unicellular, colonial, or filamentous photoautotrophic microorganisms from the kingdoms Bacteria, Plantae, Chromista, and Protozoa.^1^ These organisms are responsible for 50% of global oxygen production^2^ and are the foundation of the aquatic food chain due to their balanced and favorable amino acid and lipid profiles.^3^ Microalgae are highly productive and have gained commercial relevance due to intensive cultivation and generation of a diverse portfolio of renewable bioproducts including essential *n*-3 polyunsaturated fatty acids (e.g., eicosapentaenoic acid^4−6^), single-cell protein,^7^ biodiesel,^8,9^ cosmetics (e.g., sunscreens,^10^ moisturizers,^11^ antiaging products^12^), and antioxidants (e.g., β-carotene,^13^ astaxanthin,^14^ c-phycocyanin^15^). Further, microalgae represent the third and fourth generations of biofuels, preceded by first-generation edible food crops (e.g., wheat, rice, corn, vegetable oils) and second-generation inedible crops (e.g., lignocellulosic biomass).^16^ Microalgae have reported higher photosynthetic efficiencies than even the highest-performing C_3_ and C_4_ terrestrial energy crops,^17,18^ can leverage nonarable land for cultivation (e.g., arid regions, nonpotable water), have lower water intensities than terrestrial energy crops,^19^ and can completely utilize nutrient fertilizers.^7^

Microalgal biomass is typically cultivated outdoors in large, shallow, recirculating ponds driven by single or multiple paddle wheels, referred to as open raceway ponds (ORPs). ORP productivity (biomass produced per area per time; g/m^2^/d) is maintained by ensuring adequate water chemistry (e.g., nutrient concentrations, total dissolved solids, pH, carbon dioxide, dissolved oxygen) and by continuous or semicontinuous harvesting of microalgal biomass accompanied by dilution with fresh growth medium. Parameters including pH, total dissolved solids (TDS; g/L), dissolved oxygen (DO; mg/L), temperature (T; °C), light (measured via photosynthetically active radiation (PAR); μE/m^2^/s), and some nutrient species can be monitored *in situ* by commercially available sensors (e.g., quantum pyranometers for PAR,^20^ ion-selective electrodes for nitrate, ammonium, potassium, calcium,^21^ pH electrodes, thermistors) and coordinated with corrective actions for autonomous operation. Recently, a hyperspectral reflectance monitoring platform was developed and deployed for online detection of biomass density, quality, and even pest monitoring.^22,23^ Spectroradiometric signatures are used to calibrate the instrument against routine operating conditions (e.g., axenic algal cultures, growth medium effects). Comparative spectroradiometric profiles are then developed and evaluated against baseline conditions to identify variance in pigments, biomass densities, and the presence of pests. Taken together, these tools provide ORP operators with an immense quantity of data but no forecasting capability.

Although monitoring is inexpensive, the large volumes of water and land area occupied by outdoor ORPs make control of temperature and light cost prohibitive. This variance and lack of temperature and light control make the prediction of ORP productivity challenging as both vary both diurnally and seasonally. However, forecasting of ORP productivity is important for decision making, selection of implementation sites, and cost optimization in commercial operations. Unfortunately, outdoor microalgal processes are difficult to reliably model due to the complexity of environmental effects on photoautotrophic metabolism.^24^ On a cellular level, microalgae constantly respond and adapt to dynamic conditions that vary radically with time (e.g., pH, inorganic carbon, DO, light, T, nutrients, TDS). In terms of energy acquisition, light absorption and attenuation by cell shading and inorganic carbon availability vary over short time scales (e.g., ≪ second), whereas cellular responses including photoacclimation, pigmentation, and biomass generation occur over much longer time scales (e.g., hours to days).^25^ Due to the lack of cost-effective control mechanisms, outdoor ORPs are continuously exposed to light and temperature fluctuations, which complicates predictive modeling. Dynamic ORP models have been developed,^26−37^ but validation is rare due to abundant state variables and time-consuming model calibration since physical dimensions and operating parameters in ORP configurations can vary widely.

Separately, data-driven approaches have demonstrated the ability to model and identify nonlinear relationships in complex systems. Machine learning has demonstrated predictive capability in various research areas, including physics,^38^ materials science,^39^ chemistry,^40^ environmental engineering,^41−44^ and many more domains. However, there is only one example of data-driven modeling for ORP prediction, in which an artificial neural network (ANN) approach was applied to microalgal polyculture ORP predictions.^45^ This study was able to very accurately predict biomass density (*R*^2^_test_ = 0.94) by providing detailed inputs (e.g., initial biomass density, duration, hydraulic retention time, temperature, PAR, pH, electron donor, and nutrient concentrations); however, the experimental conditions were very controlled (e.g., contained in a greenhouse, light shading, ventilation), which is not typical of traditional large-scale ORP cultivation (e.g., outdoors, no shading, ambient ventilation). In addition, data was only collected from a single ORP in a single location (Minamisoma City, Fukushima Prefecture, Japan). Further, several input parameters used in this study, including electron donor and nutrient concentrations, require physical measurement to eliminate risk of sensor interference, which can be time and cost intensive. To improve the applicability of data-driven ORP modeling, sensors with reliable remote monitoring capabilities (e.g., pH, T, DO, PAR, TDS) could be incorporated rather than relying on time-intensive physical measurements. Further, robustness could be improved by extending data acquisition to a greater quantity of experimental ORPs and geographic locations.

In this study, we propose a data-driven, sensor profile-based deep learning approach that can outperform traditional machine learning. We capture dynamic variations in ORP conditions over time by generating parameter profile images and then develop deep learning models from these images. We apply this approach to the largest data set for ORP microalgal cultivation, the Unified Field Studies of the Algae Testbed Public-Private-Partnership (ATP^3^ UFS),^46,47^ and generate parameter profile images for five continuously monitored parameters (e.g., pH, DO, T, PAR, TDS). We then employ convolutional neural networks (CNNs) to develop predictive models for areal productivity based on these image plots. We compare our approach with a traditional machine learning approach developed from average input parameter variation throughout defined intervals (i.e., between biomass density measurements). Following initial analysis, we test the sensitivity of this new approach by modifying monitoring resolution (e.g., time between individual sensor measurements) and image resolution (e.g., total pixels per generated image) to determine performance impacts, in addition to generating and feeding synthetic data to the model to evaluate performance responses.

## 2 Methods

### 2.1 Data Set Description

The ATP^3^ UFS data set is the largest publicly available database of ORP operational data generated to date.^48^ In total, it contains >598 areal productivities and >1 M sensor records from 32 ORPs deployed in 5 U.S. states, GA, AZ, HI, CA, FL (Table 1, Supplementary Text S1, Figures S1–S8). Online monitoring provides records at a 15 min resolution; however, areal productivities were calculated from manual density measurements and therefore are only available at a 2–4 day frequency (i.e., 3 orders of magnitude more monitoring data than productivity measurements). Experimental data is available at no cost from the Department of Energy (National Renewable Energy Laboratory’s data repository, Open EI^46,47^). All data used throughout this study, including primary and secondary data manipulations, are provided here as supplementary data. Summaries of this data set are provided in Table 1, including instrumentation and productivity records delineated by the testbed site.

**Table 1:** Summary of ATP^3^ Testbed Data

			mean ± standard deviation					
ATP^3^ Testbed	Sensor Record Count	Productivity Record Count	pH	T (°C)	DO (mg/L)	TDS (g/L)	PAR (μE/m^2^/s)	Productivity (g/m^2^/d)
GA	42,858	102	8.0 ± 0.5	23.1 ± 6.4	6.8 ± 6.4	27.0 ± 4.2	377.7 ± 561.6	8.42 ± 2.79
AZ	68,127	164	8.0 ± 0.6	20.0 ± 7.2	8.0 ± 4.0	34.2 ± 3.3	526.8 ± 694.4	9.58 ± 5.04
HI	57,678	118	7.7 ± 0.6	25.8 ± 3.7	7.7 ± 3.9	33.9 ± 8.9	501.1 ± 675.3	9.55 ± 3.37
CA	54,803	117	8.0 ± 1.1	17.0 ± 6.0	8.6 ± 3.0	34.5 ± 5.3	360.3 ± 541.0	9.81 ± 3.63
FL	43,699	91	8.1 ± 0.9	25.0 ± 3.7	7.5 ± 3.3	34.3 ± 4.0	469.5 ± 673.7	8.05 ± 3.62

#### 2.1.1 Instrumentation Data

Instrumentation data was collected by Multiparameter Monitoring and Control Instruments (YSI 5200A, YSI Incorporated, Yellow Springs, OH) equipped with DO, T, pH, and TDS electrodes. A quantum pyranometer was added as an auxiliary sensor for measurement of PAR (LI-200R, LI-COR Incorporated, Lincoln, NE). Each testbed was monitored by a pyranometer (e.g., ≥1 pryanometer per testbed), and each ORP was monitored by a single YSI controller (e.g., ≥6 controllers per testbed). Sensors were installed, operated, and routinely calibrated according to manufacturer specifications (YSI, LI-COR).

#### 2.1.2 Productivity Data

Productivity was normalized on an areal basis and derived from the change in volumetric biomass density over time.^48^ Biomass density was determined three times weekly as well as prior to and following harvest/dilution operations. Briefly, known volumes of ORP culture were vacuum-filtered, dried (e.g., 95 °C), and then ashed in a muffle furnace (e.g., 540 °C) to obtain ash-free dry weight (AFDW, g/L^49^).

ORP areal productivities were calculated from eq 1, below, as
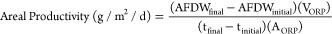
1where AFDW is ash-free dry weight (g/L), V_ORP_ is the volume of each ORP (1,025 L), t is time (d), and A_ORP_ is the area of each ORP (4.2 m^2^). Productivities were only considered during experimental harvesting periods (i.e., initial grow-out and final harvests were ignored). A further description of ORP experimentation details can be found in Text S1.

### 2.2 Data Pretreatment

Raw data was screened to provide an annual subset from the full data set. The data set was divided into four seasonal experiments corresponding to the following dates throughout 2014–2015: (a) Spring ’14: April 18–May 28, 2014; (b) Summer ’14: June 23–July 25, 2014; (c) Fall ’14: September 22–October 24, 2014; and (d) Winter ’14: December 29, 2014–January 23, 2015. Productivity data was calculated during routine harvesting operations, and final harvests were omitted from calculations (Figure S2, Text S1). Initiation of semicontinuous harvesting operations was assumed to be representative of a pseudo-steady state biomass harvest/dilution condition. In this condition, ORPs were harvested and diluted with fresh medium in defined intervals. Instrumentation outliers for each input parameter were determined by creating box plots, in which any data points that fell outside the whiskers of the box plot were determined as outliers and subsequently removed from the data set.

### 2.3 Model Development

During experimentation, instrumentation data was collected at a 15 min resolution, while biomass density was measured every 2–4 days. It is not practical to manually determine biomass density and areal productivity every 15 min since algal biomass synthesis occurs at a much slower rate than fluctuations in water chemistry. As algal productivity represents the net rate of biomass synthesis, it can be characterized by the time-series of instrumentation data. However, it varies from traditional time-series data, in which there are labels for samples at each time, such as stock price fluctuations over time. To characterize the relationship between the time-series of sensor data with biomass productivity, we proposed two approaches: (1) an average-value approach and (2) an image-based approach, both described below. Prior to model development, we randomly split the data set into training and test sets. The test set was kept the same for the average-value model (AVM) and image-based model (IBM) to guarantee fair comparison.

#### 2.3.1 Average-Value Model (Machine Learning)

The simplest approach to describe this time-series data is to characterize the given trends in instrumentation records over each productivity period by arithmetic averaging. For a given productivity measurement, we calculate the average instrumentation value of each input parameter over the corresponding time period between biomass density measurements. For example, we measure the productivity over a period of 7 days while also gathering instrumentation data for these 7 days in 15 min increments. We then take the average instrumentation data values over these 7 days as the final metric for that productivity measurement. Hence, we obtain a tabular data set for the average-value approach, in which each row contains the averaged parameters and the corresponding productivity. Following our previous studies,^39,50^ we use CatBoost to develop models for the tabular data set. Specifically, we apply 5 cross-validations on the training set and use Bayesian optimization to optimize the hyperparameters of CatBoost. After obtaining the optimum hyperparameters, we retrain CatBoost on the whole training set to obtain the final model. The predictive performance of the final model is evaluated by performance on the test set. Root-mean-squared-error (RMSE), mean absolute error (MAE), and coefficient of determination (*R*^2^) are used as evaluation metrics to evaluate model performance. The lower the RMSE and MAE and the higher the *R*^2^, the better the model performance.

#### 2.3.2 Image-Based Model

Another approach we propose is conversion of instrumentation data profile plots to images, in which the data is plotted against time to record dynamic trends, and then we employ convolutional neural networks (CNNs) to develop predictive models. Aside from capturing trends, we also benefit from a CNN due to advantages in its transfer learning and data augmentation capabilities. For each time period in which a productivity is measured, we first plot each instrumentation record (e.g., pH, T, PAR, TDS, and DO) versus time to obtain different images and then splice them together to form a 50,176-pixel image (e.g., 224 × 224 pixels). In other words, each image contains all records of instrumentation data vs time for a given productivity measurement, while one image corresponds to each productivity. In this fashion, we obtain representative images for each productivity in the training set and test set. Different from average-value based model development, we split the training set to a subtraining set and a validation set. The training set is used to train our CNN model, and the validation set is used to control overfitting. We employ early stopping to control overfitting; that is, if the predictive performance on the validation set is not increased for a certain epoch, the training process is halted. We employ DenseNet121,^51^ a specially designed architecture with fixed hyperparameters to train, which is pretrained on the ImageNet-1K data set^52^ containing over 1 M images. The validation set is not used for training and can be seen as wasting data. Traditional cross-validation can fix this issue–however, we employ transfer learning here. Since no hyperparameters need tuning, cross-validation cannot be applied here. To fully utilize the available data, we split the training set 5 times to obtain 5 subtraining sets and 5 validation sets. We train the CNN on these data sets to obtain 5 models, and then averages of the 5 models are used to evaluate performance.

#### 2.3.3 Sensitivity Analysis

Sensitivity analysis is conducted using each input parameter, monitoring resolution, and image resolution. For input parameters, we use individual images (224 × 224 pixels) of each input vs time plot to develop the models. The initial image-based model is derived from the highest monitoring resolution available, 15 min.

We then probe the modeling methodology by developing new, independent models that introduce consecutively fewer records to each model, to test the hypothesis that lower input monitoring resolution decreases the model’s ability to capture temporal trends, thereby decreasing prediction performance. First, we develop models with resolutions of 15, 60, and 240 min to establish a relationship between monitoring resolution and performance, *R*^2^_test_. Next, we verify this relationship by developing two additional models with resolutions of 120 and 180 min.

Separately, we challenge our methodology by modifying image resolution (i.e., pixel density). The original image-based model is developed with 224 × 224-pixel images, containing 50,167 total pixels. Similar to monitoring resolution, we test the hypothesis that pixel density controls performance (e.g., higher image pixel density improves prediction accuracy). However, rather than selecting from a continuous spectrum of pixel density options, we are constrained to three choices. As such, we develop two new models, each of higher image resolution. The initial resolution is improved by creating (a) 336 × 336 images, containing 112,896 pixels (e.g., a factor of 2.25), and then (b) 448 × 448 images, containing 200,704 pixels (e.g., a factor of 4).

## 3 Results and Discussion

### 3.1 Average-Value Model (AVM)

**Figure 1:**
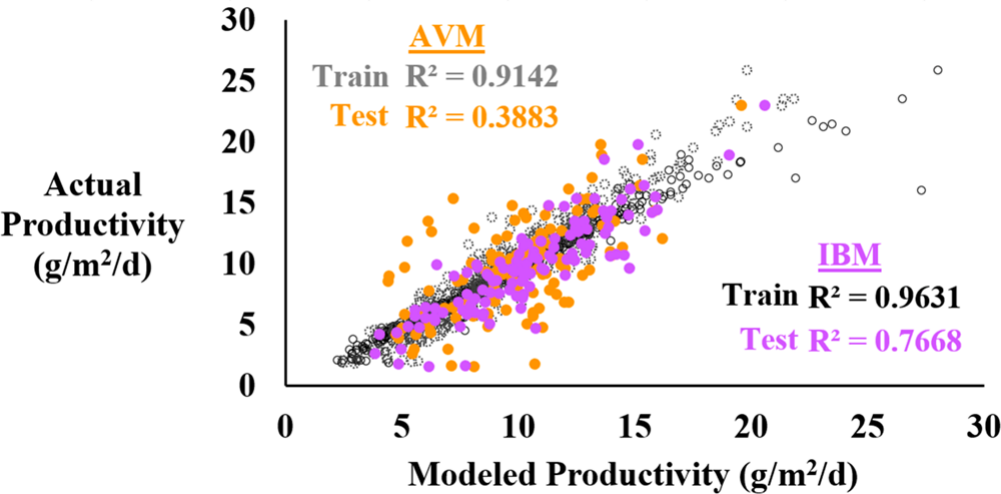
AVM vs IBM Performance Comparison. IBM (training set: black, test set: purple) outperforms AVM (training set: gray, test set: orange). Both *R*^2^_train_ and *R*^2^_test_ were significantly higher in the IBM compared to the AVM.

The Average-Value Model (AVM) performs poorly in predicting ORP productivity from the instrumentation data provided (*R*^2^_train_ = 0.9142; RMSE_train_ = 1.3547; MAE_train_ = 1.0542; *R*^2^_test_ = 0.3883; RMSE_test_ = 3.1257; MAE_test_ = 2.4711; Figures 1, S9, S13, Table S1). This approach fails to capture dynamic variations in instrumentation data due to representation as an arithmetic average. For example, the average temperature could be equal for two 7-day periods, but temperature dynamics in each scenario could vary greatly and create comparatively different growth effects. Biomass growth is nonlinear with respect to temperature^53^ and can be impacted harshly by temperature extremes. For example, a brief temperature spike to 100 °C for a short duration could cause irreparable cellular damage, eliminating the potential for future productivity. Obviously, average instrumentation data cannot adequately capture dynamic patterns for sensitive parameters. Representing the time course of instrumentation data fluctuations by a single number does little to describe the range and variance of the data trends, which are expected to significantly influence biomass growth and productivity.

### 3.2 Image-Based Model (IBM)

**Figure 2:**
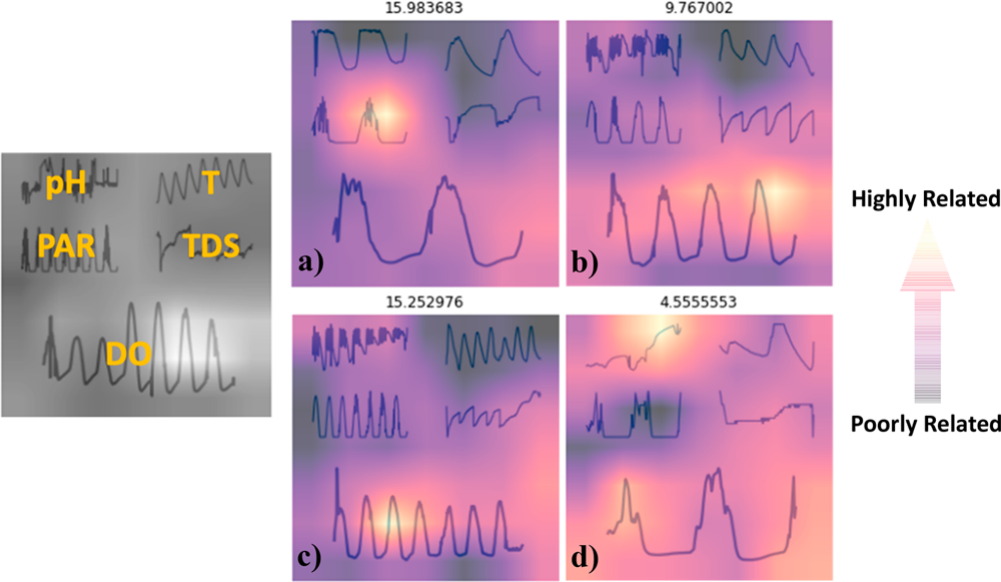
Example parameter profile plot image and IBM interpretation by Grad-CAM. Hot spots are indicated in orange/yellow as seen in the color slider (right). Hot spots suggest the IBM correlates these image locations with high influence on productivity prediction. This subset of images suggests significant relevance of PAR, DO, and pH to prediction performance. a) Cellana (HI), ORP #2, 6/23/14–6/25/14 (UFS-3); b) ASU (AZ), ORP #6, 7/14/14–7/18/14 (UFS-3); c) ASU (AZ), ORP #2, 6/30/14–7/7/14 (UFS-3); d) ASU (AZ), ORP #6, 10/8/14–10/10/14 (UFS-4).

**Figure 3:**
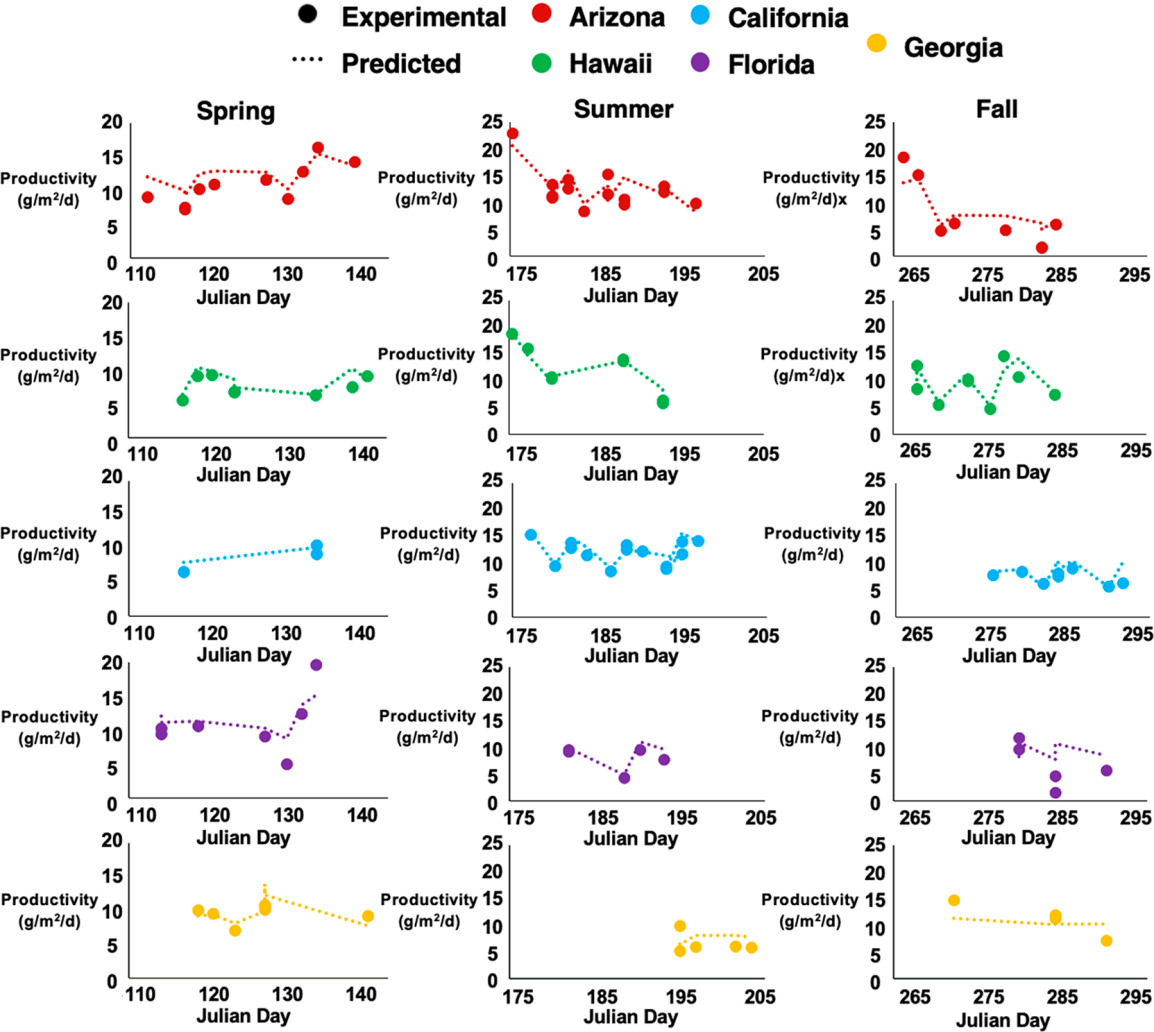
IBM time profiles. IBM test set predictions are plotted with experimental measurements across all 5 testbeds for Spring, Summer, and Fall seasons. Due to data availability and random test set selection, Winter is omitted from the plot above. The IBM is able to accurately capture productivity trends across seasons and geographic locations.

Separately, the 15 min resolution Image-Based Model (IBM) demonstrates superior prediction performance when compared to the AVM for all evaluation parameters (*R*^2^_train_ = 0.9631; RMSE_train_ = 0.8886; MAE_train_ = 0.5291; *R*^2^_test_ = 0.7668; RMSE_test_ = 1.9901; MAE_test_ = 1.5081; Figure S10, Table S1). Relative to AVM test set prediction performance, the IBM exhibits improvements of 97.5% for *R*^2^_test_, 36.3% for RMSE_test_, and 39.0% for MAE_test_. An example parameter profile plot image and a selection of Grad-CAM hot spots are provided in Figure 2. Grad-CAM hot spots were generated for all IBM parameter profile plot images, and a subset of four images is provided here to help visualize both parameter profile plots and the significance of hot spots. For context, the test set predictions are plotted and overlaid by experimental values, separated by a testbed site and season in Figure 3. The improvement in prediction performance can most likely be attributed to the detailed description of environmental and water chemistry variations over the time course of each productivity calculation that was extracted by the IBM. For example, over a 2-day interval between productivity calculations, the IBM captures 192 data points for each instrumentation parameter in comparison with the AVM which only captures one. Since the IBM clearly outperforms the AVM, the IBM was selected for further method optimization, below.

### 3.3 Effect of Monitoring Resolution on IBM Performance

The results of IBM optimization by modification of monitoring resolution are presented below (Table 2). Three monitoring resolutions were chosen, each larger than the previous by a factor of 4 (e.g., 15, 60, 240 min, Figure S11, orange). Following the initial three IBMs, two more resolutions were chosen to verify the previous relationship (e.g., 120-, 180 min, Figure S11, blue). A clear trend is observed, with predictivity, *R*^2^_test_, decreasing logarithmically with increased monitoring resolution (*R*^2^_test_ = −0.1 ln(resolution; minutes) + 1.0298; *R*^2^ = 0.9919). This is supported by increases in RMSE and MAE in the test set as well. An increase in resolution from 15- to 240 min created penalties of 36.1%, 38.6%, and 36.3% in *R*^2^_test_, RMSE_test_ and MAE_test_, respectively. This result is intuitive but not trivial. As we provide the model with less detail, represented here by fewer records, and longer durations between each record, we decrease our ability to capture temporal trends in parameter variations. As such, error increases and accuracy decreases.

**Table 2:** Results of IBM Modification of Monitoring Resolution

	Training Set			Test Set		
Monitoring Resolution (min)	*R*^2^	RMSE	MAE	*R*^2^	RMSE	MAE
15	0.9631	0.8886	0.5291	0.7668	1.9901	1.5081
60	0.9749	0.7233	0.4939	0.6069	2.5060	1.9715
120	0.9709	0.8688	0.5989	0.5921	2.5317	2.0060
180	0.9754	0.7076	0.4690	0.5476	2.6067	2.0561
240	0.9638	0.8927	0.5884	0.4902	2.7581	2.1659

Based on these results, the highest monitoring resolution, 15 min, is selected to improve accuracy and reduce error. In this study, the 15 min resolution is the highest possible, as lower increments are not available from the original data set. We speculate that increasing this value further could improve accuracy and reduce error; however, this could also increase noise that is not necessarily useful for prediction. This is an open question that should be investigated in future ORP experiments to determine the absolute optimum resolution for data-driven prediction. On the other hand, as the monitoring resolution increases, costs increase, as requirements for hardware and software grow (e.g., data storage, computational power). This trade-off should be considered prior to operational implementation.

### 3.4 Effect of Image Resolution on IBM Performance

Following confirmation that the 15 min monitoring resolution provides the highest IBM accuracy as evaluated by test set metrics (e.g., *R*^2^_test_, RMSE_test_, MAE_test_), this monitoring resolution was chosen for optimization by modifying its image resolution. IBM-generated images increase from 224 × 224 (50,167 pixels), to 336 × 336 (112,896 pixels, 2.25 times more pixel density), and finally to 448 × 448 images made up of 200,704 pixels, having 4 times more pixels than the original. Model performances are presented in Table 3 and Figure S12.

**Table 3:** Results of IBM Modification of Image Resolution

	Training Set			Test Set		
Image Resolution (pixels)	*R*^2^	RMSE	MAE	*R*^2^	RMSE	MAE
50,167	0.9631	0.8886	0.5291	0.7668	1.9901	1.5081
112,896	0.9792	0.7177	0.5353	0.7447	2.0499	1.6065
200,704	0.9801	0.6712	0.4962	0.7051	2.1838	1.6934

Here, we observe that an increase in image detail (i.e., pixel density) produces lower accuracy and higher error than the original. Increasing the detail 2.25 times, from 50,167 to 112,896 pixels, creates penalties in the test set of 2.9%, 3.0%, and 6.5% in *R*^2^_test_, RMSE_test_, and MAE_test_, respectively. When image detail is increased 4 times to 200,704 pixels, we observe penalties of 8.0%, 9.7%, and 10.9% in *R*^2^_test_, RMSE_test_, and MAE_test_. We speculate this decreased performance was created because DenseNet121 was pretrained on 224 × 224 images (i.e., transfer learning). Increasing the size of images leads to a decreased transfer learning effect, thus achieving lower predictive performance.

### 3.5 Sensitivity Analysis by Synthetic Data Processing

Akin to classical sensitivity analysis, we determine the sensitivity of each input parameter by quantifying modeling performance responses (e.g., impact to productivity prediction) from variations in input parameters. First, we select the highest performing prediction and its associated image for each of three hydraulic retention times (i.e., time between productivity measurements), 2, 3, and 7 days between harvest/dilution operations. Then we modify the input parameters from the original data set to create synthetic data that maintains a similar trend. We select two approaches: (1) to modify the vertical position of the trend while maintaining the trend itself and (2) to modify the magnitude of the trend while maintaining the vertical position. These manipulations are listed below (eqs 2, 3) as
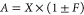
2
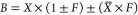
3where *A* and *B* are the synthetic manipulations from (1) and (2) above, *X* is the original parameter value, *F* is the manipulation factor (e.g., 0.05, 0.10, ..., 1.0), and *X̅* is the mean parameter value for the parameter profile plot.

However, the mechanics of the IBM create challenges to the scaling of input parameters in this fashion. For example, simple arithmetic manipulations of parameter profile plots by addition and subtraction (eq 2) yield no change to the predicted productivity. We speculate this effect is an artifact of the underlying mechanics of the IBM–the model interprets the parameter trend but is unable to discriminate between the vertical position of trends on the image plot. Next, we increase or decrease the extent of each parameter trend by multiplication and division. Multiplication (eq 3) is unable to capture variations. We speculate this is a *y*-axis scaling artifact derived from Python’s image plotting function. If the *y*-axis range is automatically determined by the extent of the data, and the *y*-axis range is greater than the original parameter profile plot range, the *y*-axis will be scaled to fit. If the *y*-axis range is less than the original, the *y*-axis is kept the same as the original. As such, only the division operation yields results, taken as eq 4, below
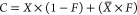
4where *C* is the synthetic data manipulation. This manipulation can be visualized in Figure S14.

**Figure 4:**
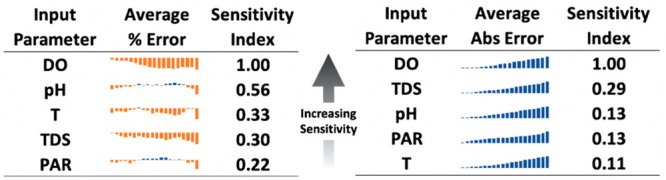
Absolute and relative parameter sensitivity with HRTs aggregated into a single data set. In terms of relative sensitivity, DO > pH > T ≅ TDS > PAR. In terms of absolute sensitivity, DO > TDS > pH = PAR ≅ T.

Following computation, synthetic data prediction performance is compared with the performance on the original data set and evaluated via percent error and absolute error. Further, a new evaluation index is created, the sensitivity index, defined by the cumulative error of a given parameter across the range in manipulation factors, *F*, (e.g., 0.05, 0.10, ..., 1.0) normalized to the cumulative error of the most sensitive parameter (i.e., the most sensitive parameter has a sensitivity index of 1.0). In this fashion, the results are provided in Figure 4 and Figure S15. In Figure 4, all hydraulic retention times are aggregated as a single data set. In Figure S15, trends in absolute error are delineated by parameter as well as hydraulic retention time (HRT).

According to the sensitivity index defined previously, relative parameter sensitivity decreases as follows: DO > pH > T ≅ TDS > PAR. In terms of absolute sensitivity, DO > TDS > pH = PAR ≅ T. DO is the most sensitive parameter in all three HRTs examined (e.g., 2, 3, 7 days). It is also the most sensitive parameter when all HRTs are aggregated into a single data set, in terms of both absolute error and relative error (i.e., percent error). Although intuitive, this result is not insignificant. DO is directly influenced by photosynthesis via oxygen evolution, and soluble concentrations are frequently observed to reach saturation during daytime photosynthesis in ORPs when microalgal photosynthesis is most active. However, DO only tends to be a strong predictor when considered along with the other 4 parameters; DO by itself does not provide superior prediction performance (IBM with DO as sole input parameter could not outperform 5 parameter AVM; unpublished). This result supports the implementation of a suite of low-cost sensors rather than investment in a single, highly sensitive sensor, such as DO, and suggests expanding monitoring instrumentation capabilities may improve prediction robustness.

### 3.6 Implications of IBM Methodology

The IBM methodology we develop throughout this study is able to accurately predict outdoor microalgal biomass productivity in ORPs despite variations in HRT, biomass density, T, PAR, DO, and TDS across five different geographic locations in the United States. The IBM outperforms traditional machine learning (AVM) and is able to nearly double prediction performance (*R*^2^_test, IBM_ = 0.7668; *R*^2^_test, AVM_ = 0.3883). Considering that biological processes are challenging to model and accurately predict, this is a notable achievement given the lack of fundamental biological parameters provided to the model (e.g., nutrient concentrations, biomass density, and hydraulic retention time). This method could facilitate commercial ORP operations to streamline downstream biomass processing, more accurately manage cost/price fluctuations, and suggest more efficient staffing, scheduling, and labor-hours. Beyond ORP forecasting, this remote monitoring-based, data-driven modeling methodology could potentially be translated to other environmental applications, including food crop productivity, drinking water and wastewater treatment processes, lake and river eutrophication, and other ambient, environmentally influenced, highly dynamic systems. The methodology presented here could facilitate valorization of large, existing, longitudinal data sets and potentially provide actionable modeling capabilities to inform technology development and deployment.

## Data Availability

Code is available at https://github.com/nogoodnameye/algal-growth

## Supporting Information Available

Raw and processed data (XLSX)
Raw and processed data (XLSX)
Text S1: Data Collection from ATP^3^ UFS Operations, Figure S1: ATP^3^ mini-pond ORP schematic, Figure S2: Example AFDW density profiles (GA), Figure S3: Productivity profile plot for 5 ATP^3^ testbeds, Figure S4: PAR profile plot for 5 ATP^3^ testbeds, Figure S5: T profile plot for 5 ATP^3^ testbeds, Figure S6: DO profile plot for 5 ATP^3^ testbeds, Figure S7: pH profile plot for 5 ATP^3^ testbeds, Figure S8: Salinity (TDS) profile plot for 5 ATP^3^ testbeds, Figure S9: AVM performance, Figure S10: IBM performance, Figure S11: Results of monitoring resolution modification, Figure S12: Image resolution modification results, Figure S13: AVM parameter correlation heat map, Figure S14: Synthetic data generated by trend dampening, Figure S15: Absolute parameter sensitivity across all HRTs, and Table S1: AVM and IBM performances (PDF)
