# Supplementary material for: Real-Time Sensor Data Profile-Based Deep Learning Method Applied to Open Raceway Pond Microalgal Productivity Prediction
Source: Environ Sci Technol. 2023 May 26;57(46):17981–9. doi: 10.1021/acs.est.2c07578 (PMC10666538; doi:10.1021/acs.est.2c07578)
Supplement: Supplementary file 3 — es2c07578_si_003.pdf [file es2c07578_si_003.pdf]

Supporting Information

Real-Time Sensor Data Profile-Based Deep Learning Method Applied to Open Raceway Pond Microalgal Productivity Prediction

Thomas Igou[1,#], Shifa Zhong[2,#], Elliot Reid[1] and Yongsheng Chen[1,*]

[1] School of Civil & Environmental Engineering, Georgia Institute of Technology, Atlanta, Georgia, 30332, United States

[2] Department of Environmental Science, School of Ecological and Environmental Sciences, East China Normal University, Shanghai 200241, PR China

[#] These authors contributed equally

*Corresponding Author

E-mail: yongsheng.chen@ce.gatech.edu; Phone: 404-894-3089

Supporting information includes 12 pages with the following text, figures, and tables as cross referenced throughout the main article:

Text S1: Data Collection from ATP^3 UFS Operations

Figure S1: ATP^3 mini-pond ORP schematic
Figure S2: Example AFDW density profiles (GA)
Figure S3: Productivity profile plot for 5 ATP^3 testbeds
Figure S4: PAR profile plot for 5 ATP^3 testbeds
Figure S5: T profile plot for 5 ATP^3 testbeds
Figure S6: DO profile plot for 5 ATP^3 testbeds
Figure S7: pH profile plot for 5 ATP^3 testbeds
Figure S8: Salinity (TDS) profile plot for 5 ATP^3 testbeds
Figure S9: AVM performance
Figure S10: IBM performance
Figure S11: Results of monitoring resolution modification
Figure S12: Image resolution modification results
Figure S13: AVM parameter correlation heat map
Figure S14: Synthetic data generated by trend dampening
Figure S15: Absolute parameter sensitivity across all HRTs

Table S1: AVM and IBM performances

Text S1: Data Collection from ATP^3 UFS Operations

Year-round ORP productivity data was generated by the ATP^3 consortium from April 18, 2014 – January 23, 2015. Each of the 5 ATP^3 ORP testbeds followed Standard Operating Procedures, Laboratory Analytical Practices [43–46] and UFS protocols [47] to standardize biomass production and harvesting practices between experimental sites. Testbeds were in Georgia, Arizona, Hawaii, California, and Florida (e.g., Georgia Institute of Technology (GT) in Atlanta, GA; Arizona State University (ASU) in Tempe, AZ; Cellana (CELL) in Kailua-Kona, HI; California Polytechnic State University (CP) in San Luis Obispo, CA; Florida Algae (FA) in Vero Beach, FL). Testbeds cultured identical organisms (e.g., *Nannochloropsis maritima* KA32) in identical ORPs and followed the same procedures for sensor implementation and maintenance, seed production, growth medium preparation, ORP operation and analysis.

A detailed description of ORPs, instrumentation, strain selection and seed-to-pond biomass scale-up is available elsewhere [48]. Briefly, each testbed operated 6 identical pilot-scale paddlewheel-mixed ORPs (1 m^3 volume, 25 cm depth, 4.2 m^2 cultivation area, **Figure S1**) inoculated with *Nannochloropsis maritima* KA32 (Cellana, HI) in a standardized marine growth medium (Modified f/2 Medium [49]; 35 g/L Instant Ocean, 70 mg/L $NO3^-$-N, 10 mg/L PO_4^{3-}-P). pH was automatically controlled at a setpoint of 6.9 ± 0.1 by dosing pressurized carbon dioxide via microbubble diffuser. Over the course of the UFS experiments, density was determined according to ash-free dry weight [50] three times weekly. ORP culture conditions were monitored continuously (e.g., pH, T, DO, TDS, PAR, 15-minute measurement resolution). Following ORP inoculation, cultures grew for a period prior to initiation of semi-continuous harvesting operations. ORPs were harvested according to pond depth (e.g., 50% volume harvest = 12.5 cm harvested / 25 cm total depth) and diluted with freshly prepared growth medium. At the end of each experiment, the total ORP volume was harvested. In this study, biomass productivities were only considered during the semi-continuous harvesting period and did not account for grow-out periods and final harvests (**Figure S2**). Further, hydraulic retention time was not considered as an input variable and all experiments were combined into a single dataset.

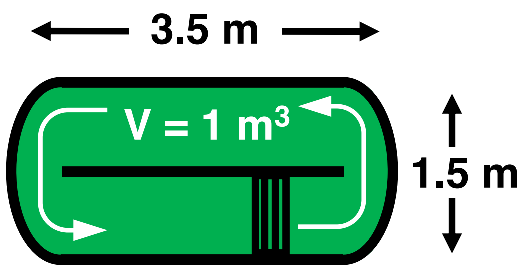

Figure S1: ATP^3 mini-pond ORP schematic. Identical ORPs were deployed at each testbed. Each testbed contained a minimum of 6 ORPs. ORPs were continuously monitored (e.g., pH, T, DO, TDS, PAR) and operated at a minimum depth of 25 cm.

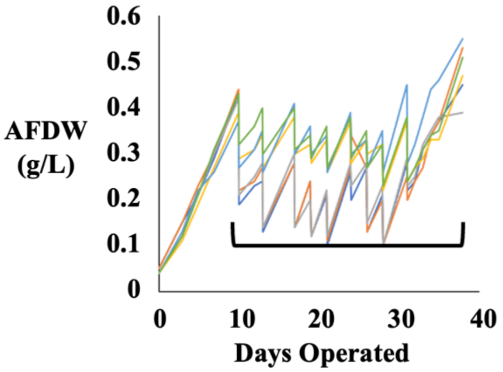

Figure S2: Example AFDW density profiles (GA). Following an initial grow-out period, ORPs were semi-continuously harvested and diluted with fresh growth medium. In this study, only results from the semi-continuous bracketed period were used.

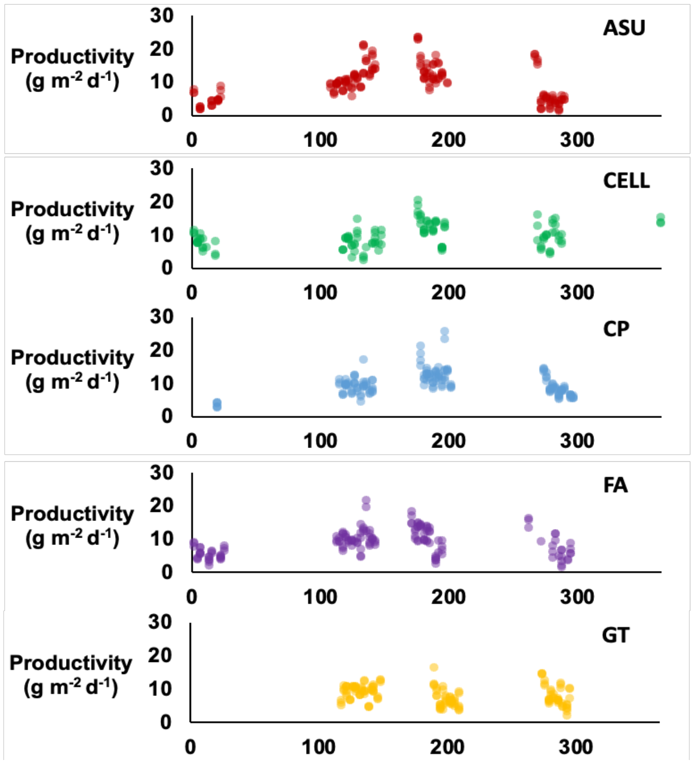

Figure S3: Productivity profile plot for 5 ATP^3 testbeds.

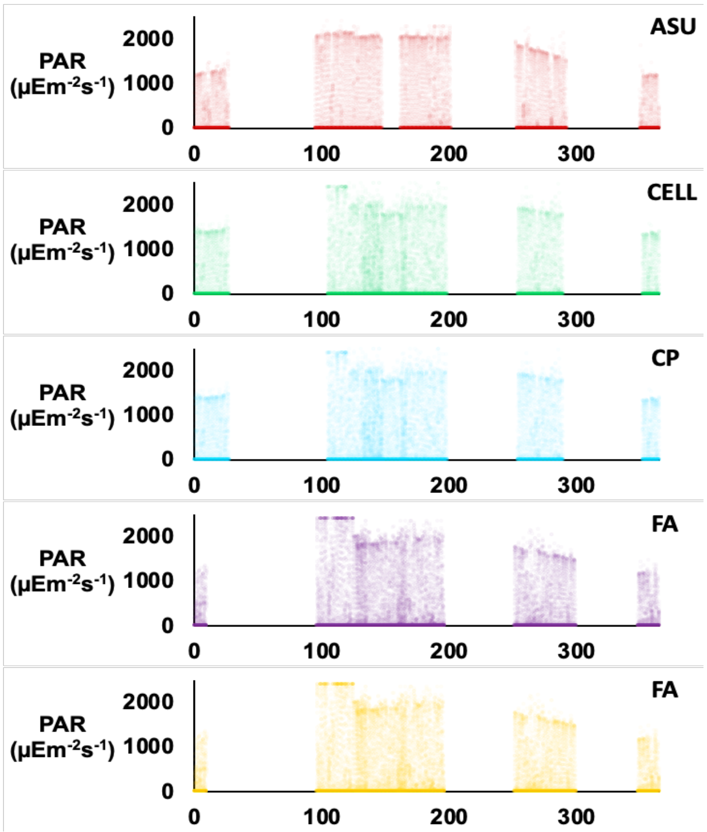

Figure S4: PAR profile plot for 5 ATP[3] testbeds.

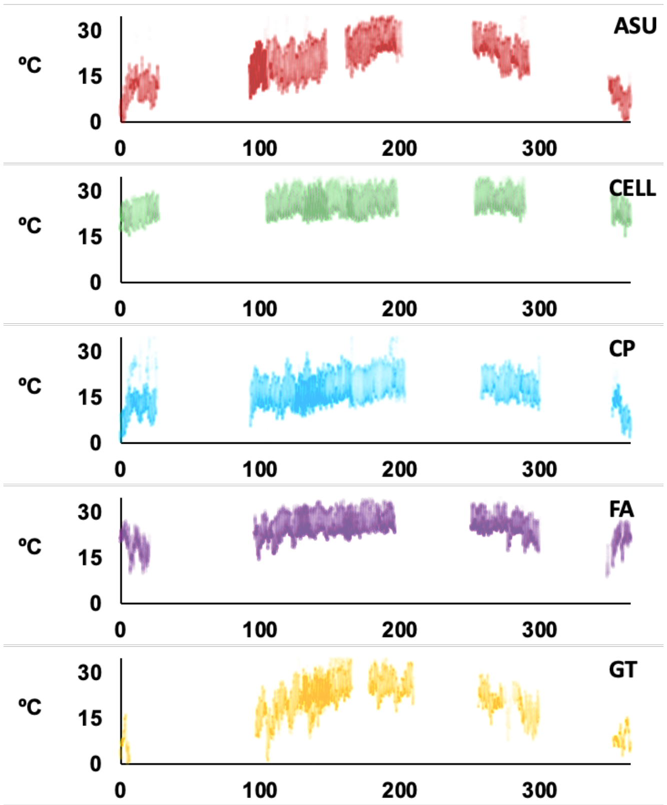

Figure S5: T profile plot for 5 ATP[3] testbeds.

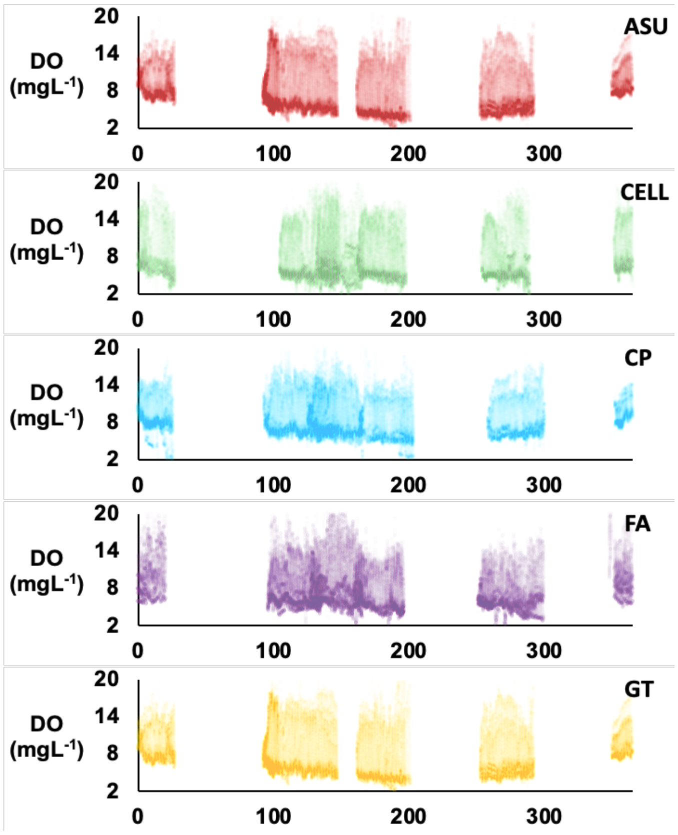

Figure S6: DO profile plot for 5 ATP[3] testbeds.

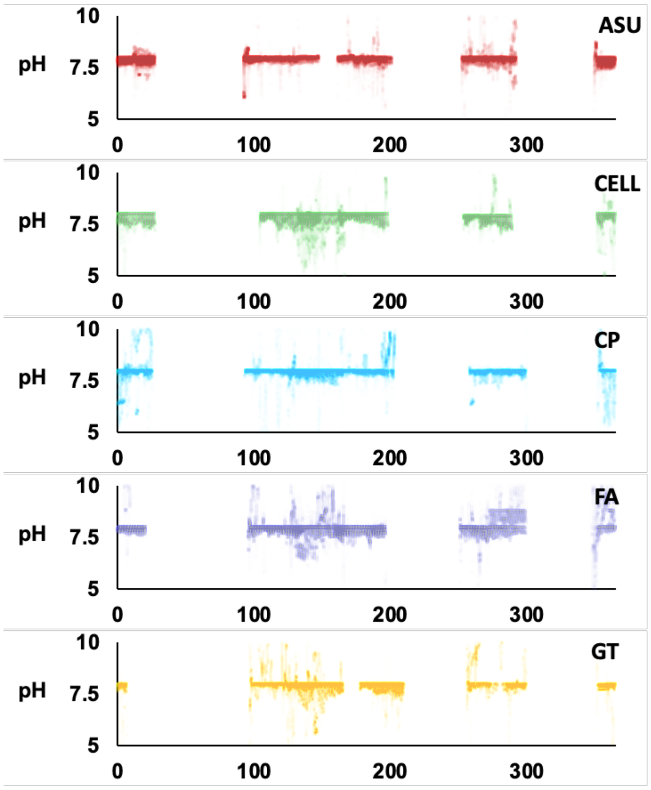

Figure S7: pH profile plot for 5 ATP[3] testbeds.

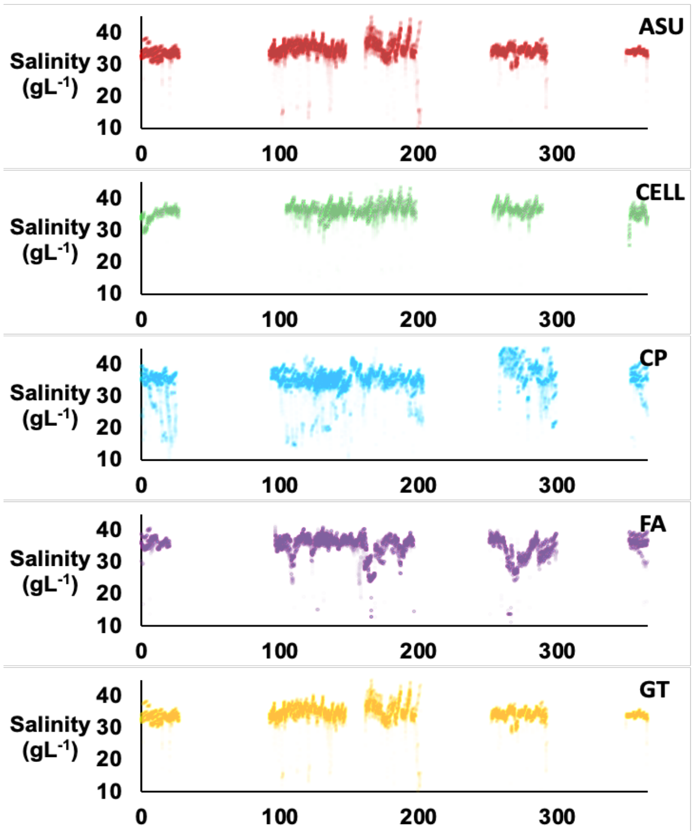

Figure S8: Salinity profile plot for 5 ATP[3] testbeds.

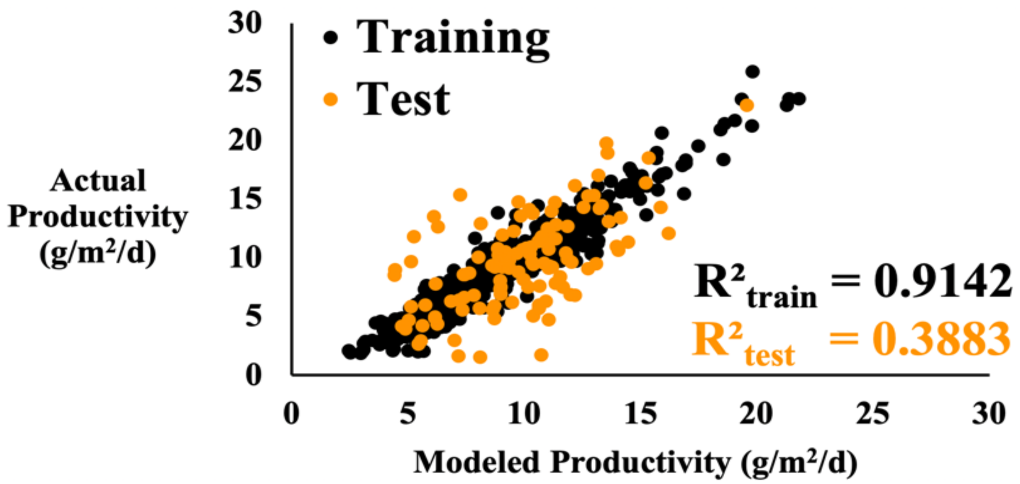

Figure S9: AVM performance. The model's performance was interpreted by linear regression of actual (y-axis) vs. predicted (x-axis) areal productivities.

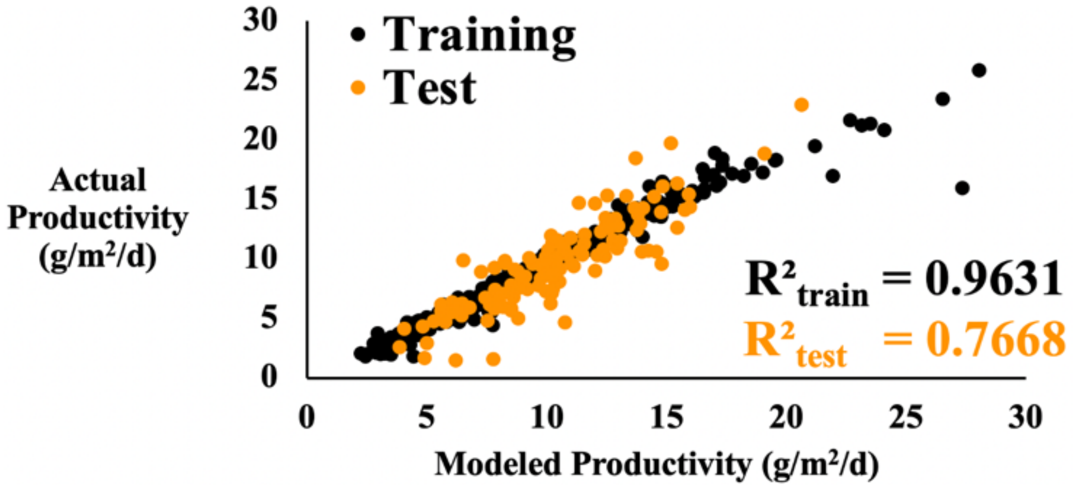

Figure S10: IBM performance. The IBM outperformed the AVM by a factor of 2. The improved performance was most likely derived from capture of detailed trends over the time course of the data in the IBM as compared to the numerical average used by the AVM.

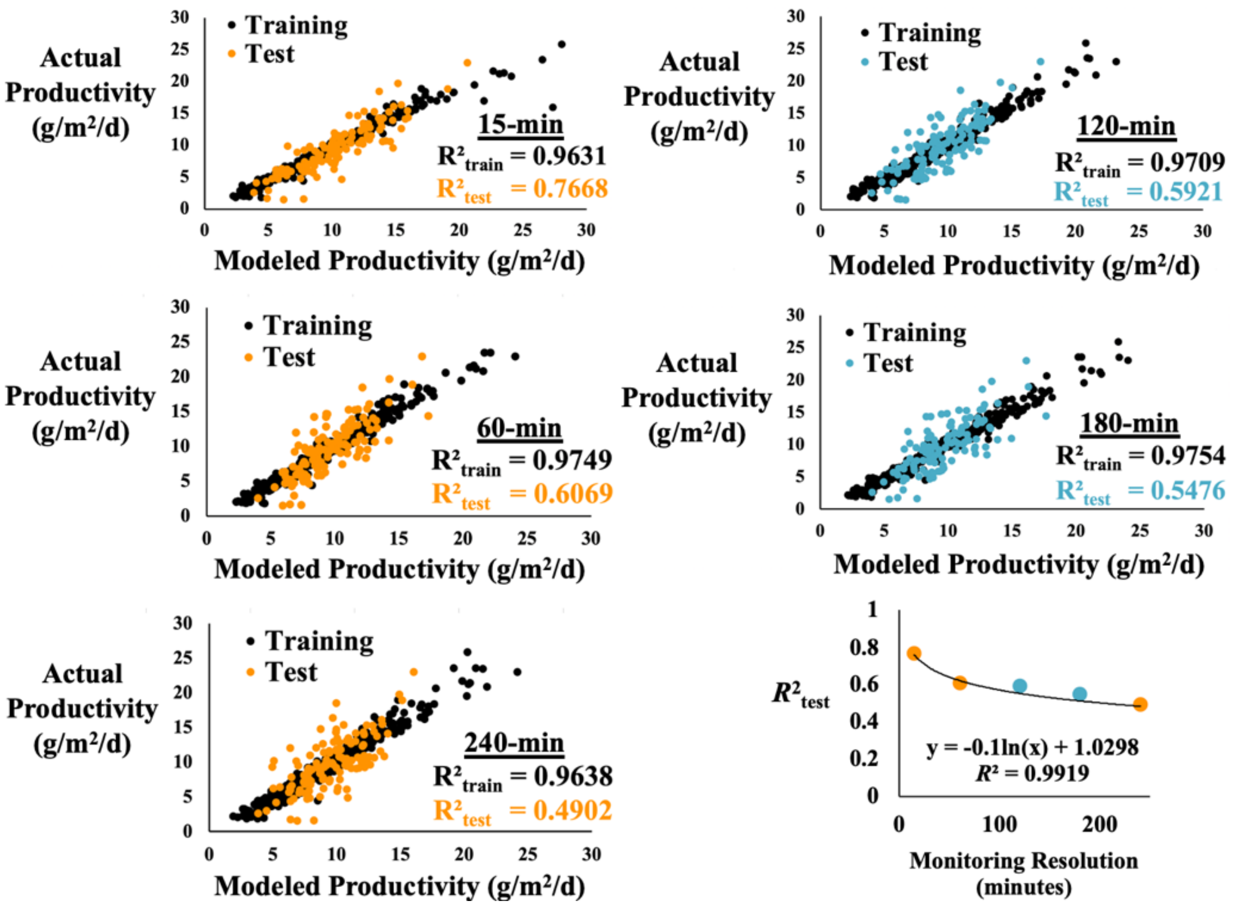

Figure S11: Results of monitoring resolution modification. Three resolutions (15, 60, 240 mins; orange) were chosen for development of an initial relationship. This was then validated by two new interpolated resolutions (120, 180 mins; blue).

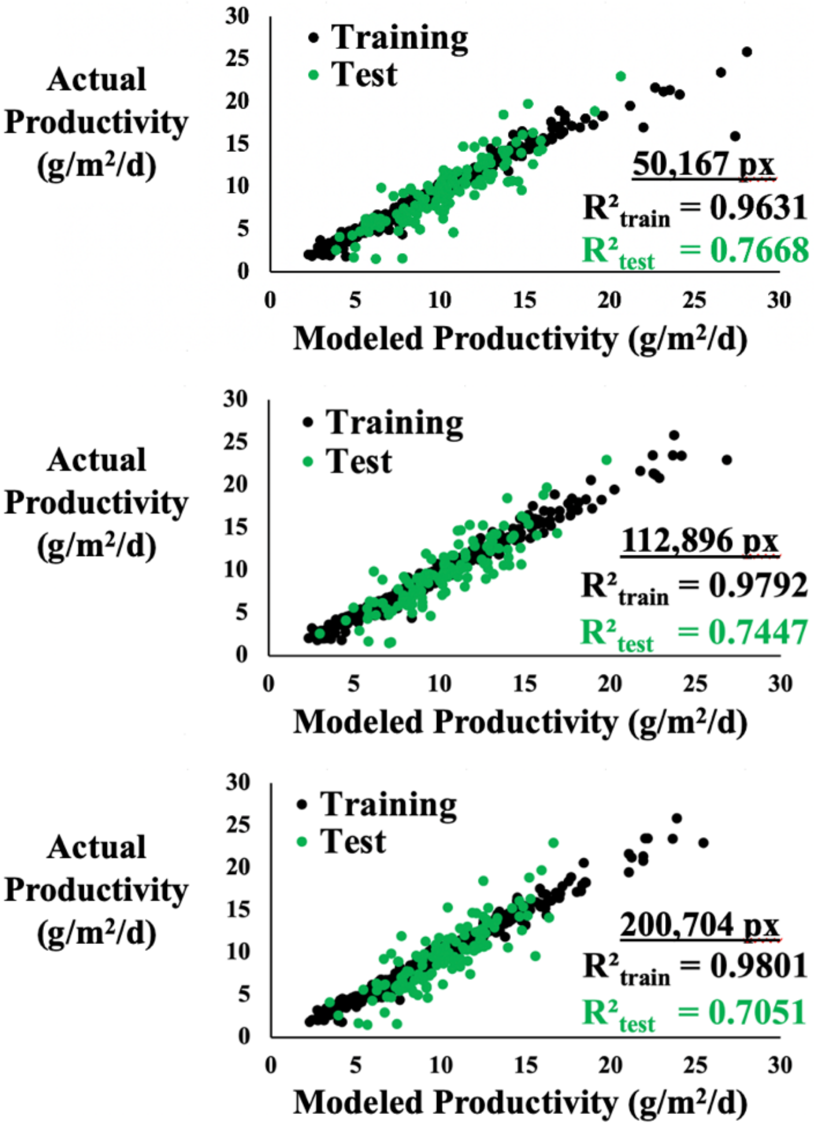

Figure S12: Image resolution modification results.

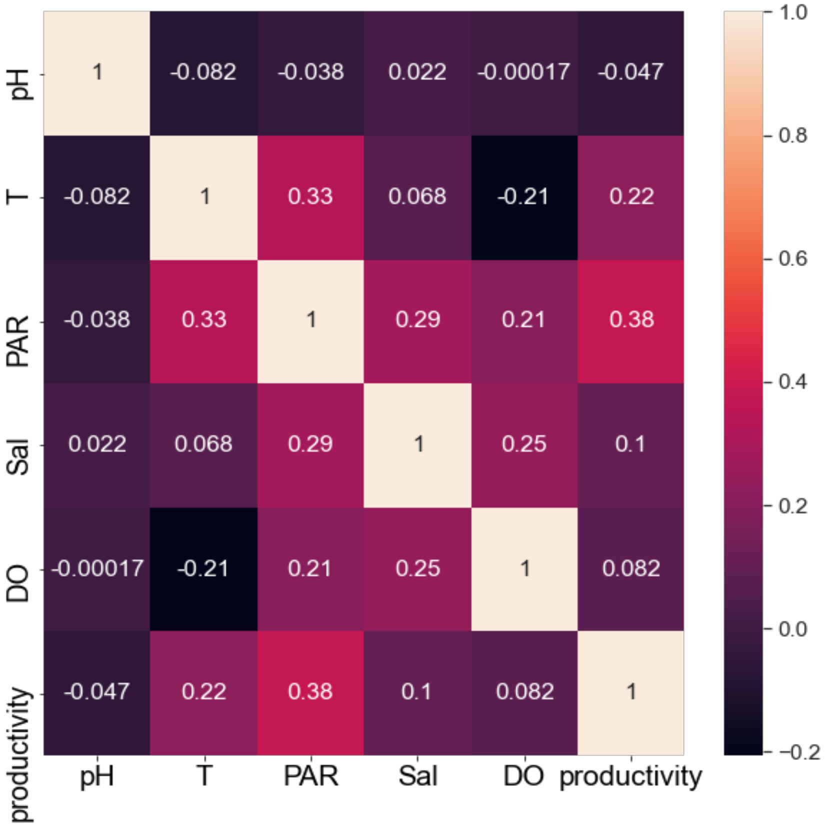

Figure S13: AVM parameter correlation heat map.

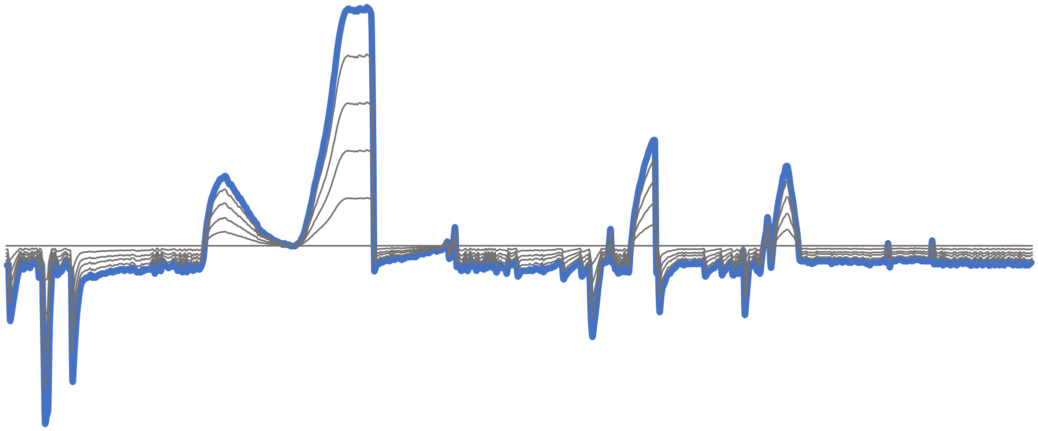

Figure S14: Synthetic data generated by trend dampening. The original trend (blue) is incrementally dampened by increasing the manipulation factor, F (Eq. 4). The resulting synthetic trends (grey) are incrementally dampened until approaching the mean parameter value, $\bar{X}$, at F = 1.0

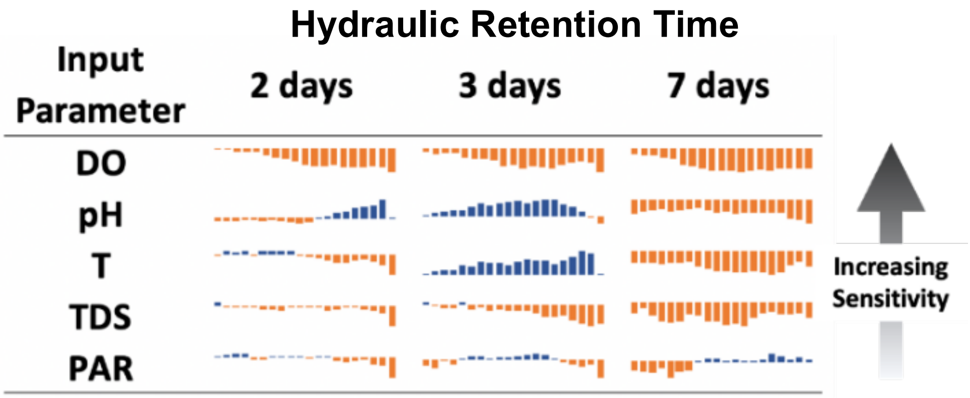

Figure S15: Absolute parameter sensitivity across all HRTs. DO is the most sensitive parameter, followed by pH, T, TDS, and PAR.

Table S1: AVM and IBM performances.

Model	Monitoring Resolution (minutes)	Image Resolution (pixels)	Training Set			Test Set		
			R^2	RMSE	MAE	R^2	RMSE	MAE
AVM	-	-	0.9142	1.3547	1.0542	0.3883	3.1257	2.4711
IBM	15	50,167	0.9631	0.8886	0.5291	0.7668	1.9901	1.5081
